# A guide for nanomechanical characterization of soft matter via AFM: From mode selection to data reporting

**DOI:** 10.1016/j.xpro.2025.103809

**Published:** 2025-05-29

**Authors:** Eunyoung Kim, Alexandra L. Ramos Figueroa, Max Schrock, Elizabeth Zhang, Christina J. Newcomb, Zhenan Bao, Lukas Michalek

**Affiliations:** 1 Department of Mechanical Engineering, Stanford University, Stanford, CA 94305, USA; 2 Department of Chemical Engineering, Stanford University, Stanford, CA 94305, USA; 3 Department of Chemistry, Stanford University, Stanford, CA 94305, USA; 4 Department of Materials Science and Engineering, Stanford University, Stanford, CA 94305, USA; 5 Stanford Nano Shared Facilities, Stanford University, Stanford, CA 94305, USA

**Keywords:** Atomic Force Microscopy (AFM), Chemistry, Material sciences, Physics

## Abstract

Atomic force microscopy (AFM) enables high-resolution mechanical characterization of soft materials at the nanoscale. It offers unique advantages over conventional mechanical testing methods by providing spatially resolved properties, requiring minimal sample preparation, and allowing measurements under controlled environmental conditions. This comprehensive guide provides a practical framework for conducting reproducible nanomechanical measurements on soft matter using AFM. Readers will learn how to select appropriate AFM modes, choose and calibrate suitable cantilevers, prepare samples, and optimize measurement parameters for soft materials. Four operational AFM modes are described: intermittent contact mode, nanomechanical imaging, force modulation, and force spectroscopy. We detail their principles, mechanisms, and trade-offs while offering practical advice for experiment execution, data analysis, and result reporting. This protocol seeks to guide researchers to execute consistent and comparable AFM measurements, bridge the gap between theoretical knowledge and practical implementation, and address key challenges in standardization and reproducibility within the field of soft matter nano-mechanics.

## Graphical abstract

**Figure:**
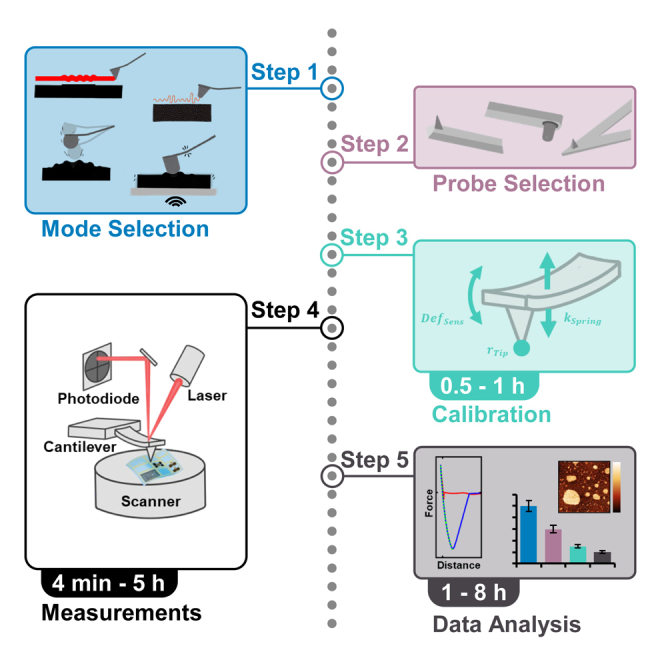

## Introduction

The invention of atomic force microscopy (AFM) in 1986 by Binnig, Quate, and Gerber marked a significant milestone in nanotechnology, enabling researchers to visualize and manipulate matter at the atomic scale.^1^ This breakthrough has driven remarkable progress across various scientific disciplines, from materials science to biology.^2^^,^^3^^,^^4^^,^^5^ The versatility of AFM has led to the development of numerous operational modes, each tailored to probe specific sample properties.^6^^,^^7^^,^^8^ At its core, AFM employs a tip attached to a cantilever that interacts with the sample surface. As the tip scans across the sample, forces between the tip and surface cause the cantilever to deflect. These deflections are typically measured using a laser beam reflected off the cantilever onto a position-sensitive photodetector, leading to high-resolution maps of topography and various surface properties, such as electrical, magnetic, chemical, and mechanical properties.^9^^,^^10^^,^^11^^,^^12^^,^^13^ This fundamental principle has made AFM an invaluable tool for characterizing a wide range of materials, including soft matter, at the nanoscale.^14^^,^^15^

Nanomechanical measurements using AFM have become a fundamental tool for characterizing the surface properties of soft matter, like polymer films, hydrogels, cells, and many other materials.^16^^,^^17^^,^^18^^,^^19^^,^^20^ These measurements offer insights into nanoscale mechanical properties of materials, ranging from thin-film technologies to bio-engineered systems.^21^^,^^22^^,^^23^^,^^24^^,^^25^ AFM has demonstrated broad applicability in characterizing biological systems and nanotechnology-related structures. For example, AFM has been extensively used to investigate viral capsids, revealing their mechanical properties and structural integrity. These insights help to understand viral assembly and infection mechanisms, shedding light on potential biomedical applications.^26^^,^^27^ Additionally, AFM has played a crucial role in mechanobiology, where it has been used to measure the mechanical properties of cells, extracellular matrices, and even intracellular components such as intermediate filaments.^28^^,^^29^ Recent advancements have also enabled AFM to probe the nano-topography of biological surfaces, such as nanogrooves that influence cellular differentiation and alignment.^30^^,^^31^ In the field of polymer science, AFM nanomechanical measurements have been employed to investigate phase separation behavior, crystallinity, and mechanical heterogeneity in thin polymer films, enabling the rational design of materials with tailored surface properties. These insights have contributed to numerous applications across different fields, for example, in applications like flexible electronics and self-healable materials.^32^^,^^33^

However, the field of nano-mechanics, particularly when applied to soft materials, faces significant challenges in terms of data reproducibility and standardization of measurement protocols. In the context of AFM measurements on soft matter, variations in sample preparation, measurement parameters, data analysis methods, and even environmental conditions can lead to significant discrepancies in results between different laboratories or even within the same research group. This lack of consistency hinders the comparison of results across studies and slows down the broader progress of the field.^34^^,^^35^^,^^36^

**Figure 1:**
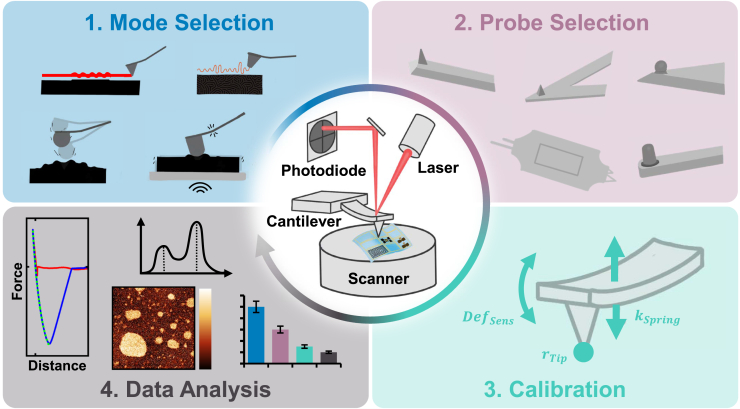
Overview of the AFM measurement process and key components of the Primer (1) Mode selection, (2) probe selection, (3) calibration, and (4) data analysis.

There are numerous reviews on nanomechanical characterization of soft matter, many showcasing specific research examples and capabilities of instrumentation, or deeply investigating theoretical physical models.^16^^,^^37^^,^^38^^,^^39^ Yet, there are only limited examples of practical, step-by-step guidance of standard procedures that can be implemented in a reproducible manner across all AFM platforms.^40^^,^^41^^,^^42^ This Primer aims to address this critical need by providing a comprehensive, practical guide to performing nanomechanical measurements on soft matter using AFM (see Figure 1). Our goal is to improve the reliability of AFM measurements and support new members of the AFM community. By offering detailed, standardized procedures for mode selection, probe pairing, instrument calibration, measurement execution, and data analysis, we seek to establish a common framework that can be adopted by researchers across the field. To facilitate implementation of these guidelines, we have included a detailed supplemental document with a step-by-step example of nanomechanical imaging measurements on a model polymer system.

## Main text

This Primer is structured around four fundamental questions that researchers often encounter when conducting nanomechanical measurements using AFM. First, we address “*What measurement technique should I select?*”, guiding readers through the selection of appropriate AFM operational modes based on their specific research needs. Further, we explore “*What probe should I choose?*”, discussing how to pair cantilevers with samples for optimal results. The third question, “*How do I calibrate my measurements?*”, discusses the crucial process of probe calibration for quantitative measurements. Finally, we tackle “*How do I evaluate the data?*”, providing insights into data analysis and interpretation. Throughout these sections, we also incorporate essential “dos and don’ts” of measuring, offering practical advice to ensure reliable and reproducible results. However, before addressing these four main questions, we want to share some sample preparation techniques.

Proper AFM sample preparation preserves sample robustness and ensures reproducible nanomechanical measurements. Typical sample substrates include silicon, glass, mica, and atomically flat gold. Mica and silicon wafers are preferred for films requiring high surface smoothness, whereas glass serves as a practical choice for thicker films. Prior to deposition, these substrates must be cleaned accordingly to remove any present contaminants. When preparing samples for AFM nanomechanical measurements, soft materials must be (1) adequately thick to prevent the underlying substrate from affecting measurements and (2) as flat as possible to allow the measurements to remain within the Z range of the instrument (typically ∼10–15 μm). As a general rule, the indentation should be <10% of the total sample thickness.^43^^,^^44^^,^^45^ Samples should be (1) uniformly dispersed across a flat substrate and (2) rigidly adhered to the substrate, and the substrate roughness must be less than the features of interest.

Polymer samples are often spin coated or drop casted. Imaging single macromolecules requires a low solution concentration before spin-coating to ensure well-dispersed features.^46^^,^^47^ For cross-sectional film imaging, samples can be prepared using a sharp blade or microtomy.^20^ Ion milling provides the highest precision for minimizing surface roughness effects but may damage samples through ion implementation and localized heat.^48^ Encapsulating polymer samples using epoxy resins can also facilitate sample handling to improve surface quality for nanomechanical measurements.

Meanwhile, imaging of biomolecules such as DNA, proteins, protein assemblies, and nanoparticles often uses surface modification of mica substrates. Common methods to promote binding between the sample and the substrate include coating surfaces with chemicals such as poly-lysine (on mica), polyethyleneimide (PEI on glass), or aminopropyltriethoxy silane (APTES on mica or silicon) to provide a positive charge for electrostatic interaction with the sample.^49^^,^^50^ The overall charge and properties of the sample need to be taken into account when choosing surface functionalization techniques. For cell culture, cells are typically grown to be sub-confluent if individual cell measurements are desired to prevent crowding and enable accurate height measurements relative to the substrate. Non-adherent cells can be challenging and require the use of microfabricated wells to physically trap them for mechanical measurements.^51^^,^^52^

Still, environmental effects need to be addressed when preparing samples, especially for oxygen- and moisture-sensitive materials. Measuring samples at consistent time points accounts for aging-related surface changes, ensuring reproducibility of surface topology and morphological properties.^53^

### What measurement technique (mode) should I select?

**Figure 2:**
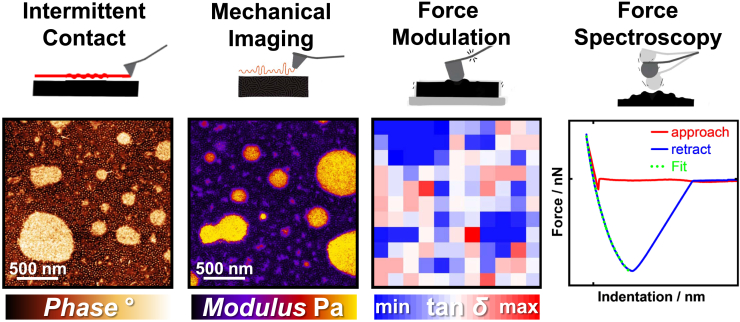
Comparison of AFM data obtained using four different operational modes on a polymer blend sample From left to right: Intermittent contact mode showing phase contrast (a qualitative assessment of mechanics), nanomechanical imaging mode displaying the modulus channel, force modulation mode illustrating loss tangent map, and force spectroscopy mode generating a force-distance curve. The color scales indicate the range of measured values for each mode. All images cover a similar 2×2 μm area of the sample, demonstrating the varying levels of detail and types of information obtainable with each mode.

AFM offers a variety of operational modes related to nano-mechanics, allowing users to choose different modes to accommodate their specific data requirements. This Primer focuses on identifying and differentiating four commonly used mechanical operational modes: intermittent contact, nanomechanical imaging, force modulation, and force spectroscopy (see Figure 2). The objective is to guide AFM users in selecting the appropriate mode based on their specific needs. Factors to consider include sample complexity, desired lateral resolution, measurement and evaluation time, and the type of mechanical properties to be investigated.

(1) Intermittent contact mode, also known as AC-, noncontact-, tapping-, dynamic-, or amplitude-modulated mode, operates by oscillating the cantilever at (or near) its resonant frequency, making intermittent (or no) contact with the sample.^54^^,^^55^ This mode is ideal for obtaining high-resolution images due to high oscillation frequencies, minimal sample interaction, and the use of sharp AFM tips with a radius of curvature <10 nm. The most useful data channels that intermittent contact mode generates for nanomechanical measurements are height and phase images. Contrast in the phase channel arises from dissipation between the tip and sample; the phase shift between the signal driving the cantilever oscillation and those experienced by the tip are monitored. For polymer systems, intermittent contact mode can distinguish between different polymer phases, as a stiffer area induces a greater phase shift, resulting in a clear phase contrast image. Unlike the other included AFM modes, intermittent contact mode requires minimal calibration, aside from determining the resonant frequency, making it a fast option for high-throughput analysis. However, its reproducibility is relatively low as the tip-sample interaction varies depending on cantilever type and imaging conditions. Other modes such as nanomechanical imaging offer more consistent results due to the use of a force setpoint that is applied with calibrated cantilevers. Therefore, intermittent contact mode is the most suitable for users seeking rapid, high-resolution topographical images to analyze the distribution of different materials, feature sizes, and surface roughness.
(2) Nanomechanical imaging applies a controlled force while in contact with the sample. By using a force setpoint and analyzing *in situ* force-distance curves, nanomechanical imaging can generate relatively reproducible and quantitative data, such as elastic mechanical properties including modulus, indentation, adhesion, and dissipation.^56^^,^^57^^,^^58^ However, nanomechanical imaging requires a more extensive calibration process, involving parameters such as deflection sensitivity, spring constant, and tip radius, which can result in longer overall data acquisition times (calibration + measurement time). The mentioned parameters are explained in more detail in later sections of this Primer. Nanomechanical imaging is particularly suitable for users who require both quantitative mechanical properties and high spatial resolution (<10 nm) of surface morphology, enabling simultaneous qualitative and quantitative comparison of different material properties.
(3) Force-modulated measurements, sometimes referred to as nano-dynamic mechanical analysis (nano-DMA), are operated by oscillating the sample or the AFM cantilever across a range of frequencies at a controlled force setpoint. It provides highly reproducible quantitative viscoelastic properties, including storage- and loss-modulus, and tan δ.^59^^,^^60^^,^^61^ As each measurement point needs to perform a frequency sweep, this mode requires significantly longer measurement times. The spatial resolution is much lower compared to the intermittent and nanomechanical imaging due to a much larger tip contact area induced by larger AFM probes, which are used to ensure good reproducibility. Force-modulated measurements are ideal for users who need more absolute and highly reproducible viscoelastic property data, particularly when sample volumes are insufficient for bulk rheological measurements. Data is often recorded at different temperatures to extrapolate relaxation times.^62^
(4) Force spectroscopy is a technique in which the cantilever tip is pressed into the sample using a linear ramp, making direct contact with the sample to obtain force-distance measurements. The force-distance curves generated during the approach, contact, and retract phases from the sample provide quantitative data, including stiffness, plastic deformation, and adhesion forces.^63^ This single-point force spectroscopy is valuable for studying local mechanical properties, making it ideal for reproducibly analyzing soft matter. While single-point force spectroscopy can be performed quickly, obtaining force spectra across an entire sample requires significantly longer measurement time compared to nanomechanical imaging. Although force spectroscopy does not produce high-resolution images due to the often larger probe tip, it offers more consistent and reproducible quantitative data. Therefore, force spectroscopy is suitable when measuring precise, localized mechanical properties, such as for heterogeneous soft materials and composites. However, due to direct contact with the sample surface, an increased risk of tip contamination can sometimes affect the measurement reliability.

**Figure 3:**
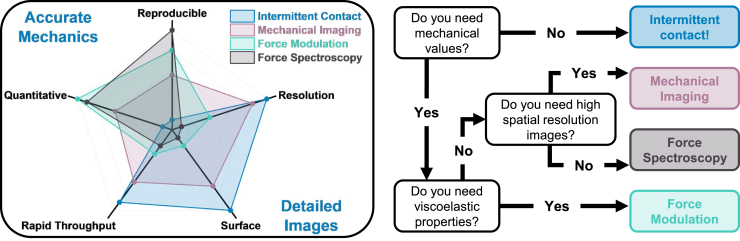
Comparative analysis of the four introduced AFM operational modes illustrating the key features, advantages, and limitations including the following factors: Spatial resolution, indentation depth from surface to sub-surface, measurement and evaluation time, data type (quantitative vs. qualitative), and reproducibility Color-coded areas are used to visually rank each mode’s performance across the factors, helping researchers quickly identify the most suitable mode for their specific experimental needs.

Selecting the most suitable AFM mode for your measurements depends on several factors, including the desired information and sample properties. You should consider the following when choosing a mode: (1) the type of data required (e.g., qualitative vs. quantitative), (2) the nature of the sample (e.g. heterogeneity, surface roughness, adhesion), (3) the desired spatial resolution, and (4) the acceptable level of tip-sample interaction (e.g. indentation). Intermittent contact mode is ideal for fast, high-resolution topographical imaging with minimal sample disturbance. Nanomechanical imaging offers a balance between spatial resolution and quantitative mechanical mapping. Force modulation excels in providing detailed viscoelastic properties but at lower spatial resolution. Force spectroscopy is ideal for precise, localized mechanical measurements. Figure 3 provides a visual comparison of these modes, highlighting their strengths and trade-offs to aid in mode selection based on specific research needs, and a simple decision tree supporting users with the mode selection.

An important parameter across all AFM modes is the setpoint, though its definition and optimization vary between techniques. In intermittent contact and nanomechanical imaging (amplitude-modulated measurement modes), the setpoint refers to (1) the target amplitude of oscillation, typically set to 70%–90% of the free amplitude, and (2) force setpoint, applied by an off-resonance frequency via multiple feedback loops (to control and monitor harmonics, force control, and time-resolved parameters for better tip-sample interaction control). For contact-based modes (direct deflection reading) like force spectroscopy and force modulation, the setpoint represents the trigger force that determines when to stop the z-piezo approach. When optimizing setpoints, a systematic approach should start with conservative settings followed by gradual adjustment of forces and feedback gains until tip-sample interaction is achieved. For detailed optimization procedures for nanomechanical imaging, see supplemental information.

### What AFM probes are there and how do I pair them with my sample?

After selecting the appropriate AFM mode for your specific research needs, choosing the right AFM probe is the next step for obtaining accurate and reliable nanomechanical measurements, especially when working with soft materials. The probe selection depends on various factors, including the sample properties, measurement mode, and desired resolution. Here we discuss the two main considerations for optimizing the probe selection for the desired measurement mode: cantilever stiffness and tip radius and shape.

(1) The cantilever’s stiffness (spring constant) should closely match the sample’s stiffness so that the cantilever deflects appropriately; a cantilever that is too stiff will not bend enough and may penetrate the sample surface, and a cantilever that is too soft will not indent the material sufficiently and cannot provide quantitative nano-mechanics. Cantilever stiffness is the primary consideration when choosing an AFM probe for nanomechanical studies. For soft materials, softer cantilevers are preferred to avoid sample damage and achieve better force sensitivity. A common practice is to choose a cantilever with spring constant similar to or slightly lower than the effective spring constant of the sample-tip interaction,^64^ typically ranging from 0.01 to 5 N/m (see Table 1). It is worth noting that even when working with soft samples, stiffer probes may sometimes be necessary if the sample exhibits strong adhesion forces, as softer cantilevers can be susceptible to sticking to the surface, preventing reliable measurements. For intermittent contact mode, probes are typically stiffer to achieve a higher resonance frequency, which allows faster scanning and reduced noise in the measurements. Therefore, when selecting cantilevers for intermittent contact mode, stiffer probes are often chosen with a spring constant in the range of 5–50 N/m, despite working with soft samples.

**Table 1:** Typical AFM probe parameter ranges for different measurement modes

AFM mode	Spring constant (N/m)	Resonance frequency (kHz)	Tip radius (nm)
Intermittent contact	∼5–50	∼150–400	∼1–10
Nanomechanical imaging	∼0.1–5	∼15–150	∼1–20
Force modulation	∼1–40	∼40–200	∼8–100
Force spectroscopy	∼0.05–15	∼6–150	∼50–5,000
(2) The geometry of an AFM tip plays a role in how forces are distributed between the tip and the sample. This force distribution is intimately linked to the effective contact area between the tip and the sample surface. Sharper tips, with a smaller radius of curvature (1–5 nm), offer high lateral resolution but may increase local stress on the sample and lead to inconsistent mechanical measurements. Blunter probes with large radii (>>10 nm) provide averaged and reproducible measurements of the nanomechanical soft matter properties, albeit with poorer spatial resolution. Colloidal probes—tips modified with microspheres of well-defined size and geometry—can be particularly useful for force spectroscopy measurements, as they provide averaged mechanical properties over larger contact areas, reducing local variations and improving measurement reproducibility.^65^^,^^66^ The contact area not only affects force measurements but also the calculation of mechanical properties such as elastic modulus. It’s important to note that the contact mechanics models used to extract mechanical properties from AFM data assume specific tip geometries in their calculations (see Box 1). Therefore, the accuracy of these mechanical property calculations depends heavily on how well the actual tip geometry matches the assumptions of the model being used. In terms of material, common choices for AFM tips include silicon or silicon nitride. Generally, silicon tips can be etched to a sharper radius of curvature than silicon nitride, but silicon nitride is often used for the cantilever to offer a softer spring constant. For specific applications that require different interaction properties (tailored adhesion), tips coated with materials such as diamond-like carbon or gold may be used.^67^^,^^68^^,^^69^
  Box 1 **Contact mechanics models in AFM nano-mechanics**
  Contact mechanics plays a crucial role in interpreting AFM measurements for soft materials. Three principal classical theories (see Figure 4)—Hertz, Johnson-Kendall-Roberts (JKR), and Derjaguin-Muller-Toporov (DMT)—are commonly used to interpret AFM data and determine mechanical properties.^70^ The Hertz theory (1882) models the contact between two linearly elastic spheres, neglecting surface forces and adhesion F=0.^71^^,^^72^ It serves as the foundation for more complex models but is limited in its application to real-world scenarios involving soft materials, as adhesion is very common. The JKR theory (1971) extends the Hertzian model by considering adhesion only within the contact region of two spheres while neglecting longer-range interactions outside contact.^73^ The incorporated adhesion can be calculated as $F=3/2\cdot \pi \gamma R^{*}$, where γ is surface energy and $R^{*}$ is the reduced radius. This model is better suited for soft and highly adhesive materials like tissue, hydrogels, and other solvated polymers. The DMT theory (1975) takes a different approach, extending the Hertzian model by considering an elastic sphere against a rigid plane surface.^74^^,^^75^ It includes the effect of adhesion $F=2\pi \gamma R^{*}$ at the interface and van der Waals forces outside the contact region. This model is more suitable for materials with minor adhesion. Choosing the appropriate model for AFM analysis can be challenging, as it depends on the material properties and experimental conditions. To address this, researchers developed the Tabor parameter μ, a dimensionless quantity that compares adhesive and elastic forces to help determine the most suitable theoretical model for a given contact situation.^76^ Understanding these models and their applicability is crucial for accurate interpretation of AFM data in nanomechanical studies of soft materials, particularly polymer films.^77^^,^^78^

**Figure 4:**
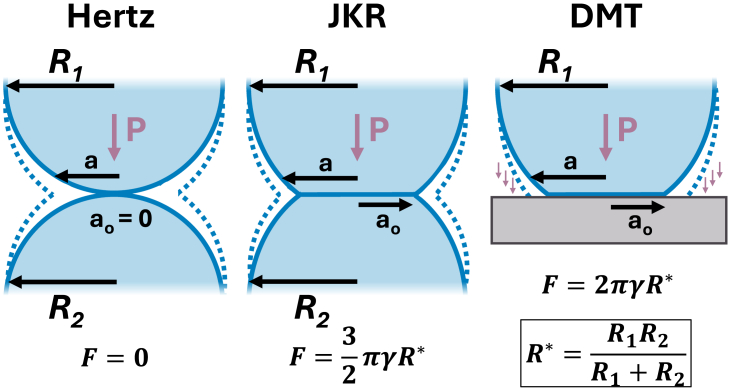
Schematic representation of three principal contact mechanics models used in AFM nano-mechanics: Hertz, Johnson-Kendall-Roberts (JKR), and Derjaguin-Muller-Toporov (DMT) The diagrams illustrate the differences in how each model treats the contact radius (a and a_0_ – radius at zero load) and adhesion forces between two elastic spheres (AFM tip) and a flat surface (sample). The Hertz model neglects adhesion forces *F*, the JKR model accounts for short-range adhesion within the contact area, and the DMT model considers long-range adhesion forces outside the contact area. Figure redrawn from O’Rorke et al.^70^

Simple contact mechanics models can be used to pair sample stiffness to an appropriate cantilever spring constant (for quantitative measurements) and tip radius. The step-by-step procedure is detailed below, and typical ranges of cantilevers can be found in (Table 1):

(1) Estimate sample stiffness: Determine the sample’s Young’s modulus (E) range from literature or bulk measurements. For many soft materials, E can range from 10 kPa up to 1 GPa.
(2) Calculate effective sample stiffness: Use Hertz model to estimate effective sample stiffness $k_{eff}\approx 2Ea$, where a is the contact radius.^79^
(3) Match cantilever stiffness: Choose a cantilever with a spring constant (k) similar to or slightly higher than $k_{eff}$. This ensures good force sensitivity without compromising stability.
(4) Consider adhesion: For very sticky samples, you may need to use stiffer cantilevers to overcome adhesion forces and achieve stable imaging or force measurements.
(5) Evaluate viscoelasticity: Many soft materials exhibit viscoelastic behavior. For dynamic measurements, consider how the cantilever’s resonance frequency compares to the characteristic relaxation times of the soft materials.

### How do I calibrate my probe (for quantitative results)?

Calibrating an AFM probe is a critical step to ensure quantitative and reliable nanomechanical measurements. Calibration of AFM probes can be relative or absolute. Relative calibration focuses on consistency between measurements, offering quicker implementation and enabling comparative studies but lacks absolute values and limits cross-setup comparability. Absolute calibration determines absolute property values, enabling quantitative comparisons across different experiment types and instruments. This calibration type is essential for standardization and reproducibility in nanomechanical measurements; however, it is time consuming (approximately an hour of extra measurements) and more prone to cumulative errors from multiple calibration steps. The choice between relative and absolute calibration depends on the specific requirements of the experiment and the desired level of quantitative accuracy. The process involves determining several probe-specific parameters. It is important to note that there is no need for extensive calibration for the “standard” intermittent contact mode measurements as they are not used for quantitative comparisons. Below is a discussion of specific calibration parameters:

(1) Spring constant (k) of the cantilever is fundamental to quantitative force measurements. It relates the applied force to the cantilever deflection and is typically measured in N/m. Accurate determination of the spring constant (rather than utilizing the nominal spring constant provided by the manufacturer) is crucial for converting deflection measurements into force values.^80^ Methods to determine the spring constant include the following:
  (a) *Thermal tuning method*: This non-destructive technique uses the cantilever’s thermal noise to calculate its spring constant. It is based on the equipartition theorem, which relates the cantilever’s mean-square deflection to its spring constant and temperature.^81^^,^^82^
  (b) *Sader method*: This method is based on the cantilever’s plan view dimensions, material properties, and resonance frequency in air.^83^^,^^84^
  (c) *Reference spring method*: This involves pressing the cantilever against a calibrated reference cantilever with a known spring constant.^85^
(2) Deflection sensitivity converts the photodetector signal (usually in volts) to actual cantilever deflection (in nanometers), often referred to as inverse optical lever sensitivity (InvOLS). It is typically expressed in nm/V.^86^^,^^87^ To determine the deflection sensitivity, measure a force curve on a hard, non-deformable surface (e.g., silicon wafer, sapphire, or mica) and measure the slope of the force curve in the contact region. In combination with the spring constant, we can calculate the force that is exerted on the sample: F[N]=k[N/m]·InvOLS[m/V]·Deflection[V]
(3) Tip area is crucial for converting force measurements into stress values. It is challenging to measure directly due to the tip’s nanoscale dimensions. Accurate tip area estimation is particularly important for force spectroscopy experiments. Methods for determining the tip radius include the following:
  (a) *Direct imaging* through other imaging methods like electron microscopy.
  (b) *Tip reconstruction*: This method uses a mathematical algorithm to reconstruct the tip shape from AFM images using a reference sample with sharp features or sample with known geometry.^88^ This procedure can be performed *in situ* without removing the tip from the instrument. The tip shape is inferred from the resulting image.
  (c) *Nanoindentation*: By performing indentations on a sample with known elastic modulus, it is possible to extract the tip area through inverse analysis.^89^ Recent developments also show the possibilities of determining the tip radius and modulus of soft matter solely based on indentation experiments.^90^

To ensure reliable and reproducible nanomechanical AFM measurements (see Box 2), a systematic calibration procedure following these key steps is essential:

(1) Determine the cantilever spring constant.
(2) Measure the deflection sensitivity on a hard, flat surface.
(3) Estimate the tip area through imaging or indirect methods.
(4) Perform test measurements on well-characterized samples to validate the calibration and potentially refine.
(5) Regularly check and recalibrate the probe, especially after prolonged use or environmental changes.

Box 2 **Dos and don’ts for nanomechanical AFM measurements**

**Dos**

Sample preparation:

• Ensure samples are clean, mostly flat, securely mounted, and free from contaminants.
• Allow samples to equilibrate to room temperature before measurement.

Measurement parameters:

• Start with conservative settings (slow scan rate, low setpoint) and optimize as needed.
• For intermittent contact mode, adjust drive to improve and evaluate phase contrast
• For very thin samples, ensure minimal indentation depth to reduce substrate effect.
• Adjust scan size, resolution, and force setpoint based on sample properties and measurement requirements.
• Use appropriate models for data fitting (e.g., Hertz, JKR, DMT) based on sample properties and tip-sample interactions (see previous note).

Data collection:

• Collect enough data points for robust statistical analysis (sufficient evaluation of standard deviation and error), especially for force spectroscopy.
• Save raw data along with processed results.
• Record all experimental parameters (e.g. set point, scan rate) and environmental conditions.
• Report uncertainties and confidence intervals with your results.

Quality control:

• Periodically verify calibration, especially for long experiments or after changing environmental conditions.
• Regularly perform measurements on a reference sample to ensure consistency.
• Check tip integrity by imaging a standard sample or obtaining force curves on a reference material.
• Watch out for sudden value changes in channels while scanning. These changes may indicate tip contamination and the tip may require replacing or cleaning.^103^

**Don’ts**

Measurement practices:

• Don’t apply excessive force, especially on soft or delicate samples.
• Avoid rapid lateral movements when the tip is in contact with the sample.
• Be aware that reflective samples/substrates can interfere with the laser signal. This interference can be minimized by selecting a wider probe.

Data analysis:

• Don’t overlook the substrate effect; modulus values can be much larger due to indirect measurement of the substrate. A deviation is expected especially for very thin films.^104^^,^^105^
• Avoid using inappropriate contact mechanics models for your sample type, especially when high adhesion forces are recorded.

Reporting:

• Don’t report results without considering and stating measurement uncertainties.
• Avoid comparing data sets obtained under different environmental conditions without accounting for these differences.

Calibration:

• Don’t assume calibration values remain constant over time or between experiments.
• Avoid using uncalibrated probes for quantitative measurements.

### How do I analyze and report the data?

Understanding and effectively analyzing AFM data is critical for accurate interpretation and reporting of nanomechanical measurements. Each measurement mode produces distinct types of data, providing different information about sample properties. Let us explore the data outputs for each mode and discuss analysis and reporting strategies.

Intermittent contact mode primarily generates two types of data: height and phase images. The height image provides topographical information, revealing surface features and roughness. The phase image offers contrast based on material properties, often a qualitative distinction between different components in heterogeneous samples.

Nanomechanical imaging mode produces a richer dataset, often including quantitative maps of elastic modulus, adhesion, deformation, and dissipation. The elastic modulus map, often referred to as the stiffness/modulus channel, provides spatial information about sample elasticity. The adhesion channel quantifies tip-sample interaction forces, while the deformation channel shows the extent of sample indentation under the applied force. The dissipation channel relates to the energy lost during the tip removal from the sample surface. The differences in contrast between some of these data channels can give insight into different surface and sub-surface properties.^91^

Force-modulated measurement generates data on viscoelastic properties as a function of frequency. Data channels include storage modulus *E′* (representing elastic behavior), loss modulus *E′′* (indicating viscous behavior), and loss tangent *tan δ* (the ratio of storage to loss modulus). These parameters are typically presented as frequency-dependent plots or maps showing spatial variations across the sample surface.

Force spectroscopy produces individual force-distance curves at specific sample locations. These curves provide detailed information about local mechanical properties, including adhesion forces, elasticity, and plastic deformation. When performed at multiple points across a sample (force volume mapping), it generates a multi-dimensional dataset that can be analyzed to create property maps similar to those obtained in nanomechanical imaging mode. For samples with large heterogeneity, the acquisition of more data points is necessary to generate statistically significant quantitative values.

**Figure 5:**
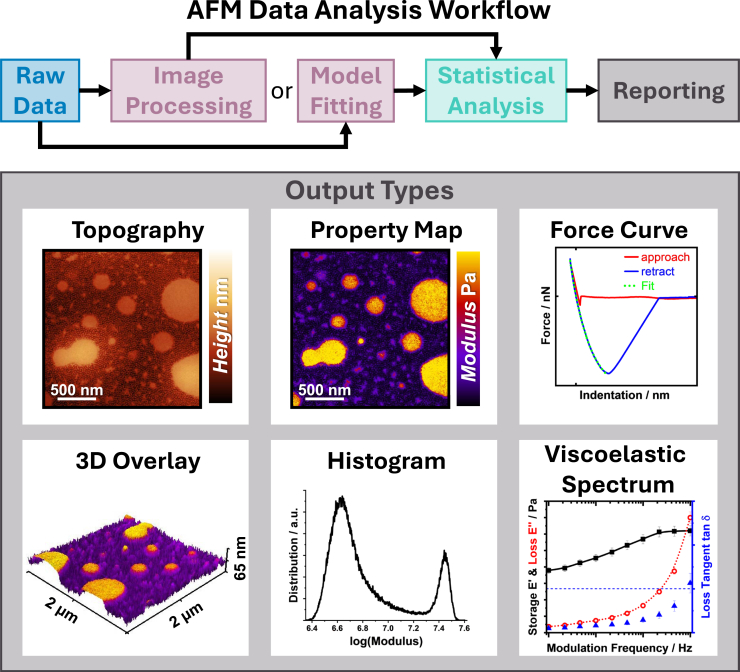
AFM data analysis workflow for nanomechanical measurements The flowchart illustrates the typical steps from raw data acquisition to final output types. Key stages include initial image processing, model fitting, and statistical analysis. Example outputs are shown, including a topography map, a 3D overlay of mechanical properties (like phase in intermittent contact mode or other data channels in nanomechanical imaging), a modulus map, a histogram of measured properties, a force curve, and a viscoelastic spectrum.

Regardless of the AFM mode used, several common analysis techniques can be applied to extract meaningful information from the data. A flow chart and example results can be seen in Figure 5.

(1) *Image processing*: For all modes that generate 2D maps or images, basic image processing techniques are essential. This includes adjusting contrast and brightness, applying appropriate color scales, and performing necessary leveling or flattening operations.^92^^,^^93^ However, care must be taken to avoid introducing artifacts or changes in absolute values, especially when processing quantitative data channels like modulus or adhesion maps.
(2) *Statistical analysis*: Histogram analysis is particularly useful for quantitative channels to identify different phases or components in heterogeneous samples. For force spectroscopy data, statistical analysis of multiple force curves can provide insights into the distribution of mechanical properties across the sample.^94^^,^^95^
(3) *Cross-correlation analysis*: Comparing different data channels (e.g., height vs. modulus or adhesion vs. dissipation) can reveal relationships between topographical and mechanical properties or can even reveal sub-surface differences. This is particularly valuable in nanomechanical imaging.
(4) *Frequency analysis*: Specific to force-modulated measurements, analyzing the frequency dependence of viscoelastic properties can provide insights into material behavior across different time and temperature ranges.
(5) *Model fitting*: For force spectroscopy and nanomechanical imaging data, fitting appropriate contact mechanics models (e.g., Hertz, DMT, JKR) to the force-distance curves is important for extracting quantitative mechanical properties. The choice of model should be justified based on the sample properties and experimental conditions.

When reporting AFM nanomechanical data, comprehensive information about the measurement conditions, data processing, and analysis methods is essential. This ensures reproducibility and allows for meaningful comparison with other studies. Key elements to include in your report are as follows:

(1) *Experimental details*: Specify the AFM mode used, probe characteristics (manufacturer and model, spring constant, resonance frequency, tip radius), and relevant operational parameters (e.g., scan size, force setpoint, oscillation amplitude, scan rate). Also, the AFM model and controller model should be specified.
(2) *Calibration procedures*: Describe the methods used for cantilever calibration and tip characterization. For quantitative measurements, report the calibration standards used and any assumptions made in the calibration process.
(3) *Sample preparation*: Provide details on sample preparation methods, as these can significantly influence the measured properties.
(4) *Data processing*: Clearly describe any image processing steps applied, including flattening or filtering operations. For quantitative analysis, specify the software used (Box 3) and any data fitting procedures or models applied.
  Box 3 **Data processing software**
  Several software packages are commonly used for analyzing AFM nanomechanical data, each offering unique features and capabilities. *Gwyddion* is a popular open-source option, providing a wide range of tools for scanning probe microscopy data visualization and analysis, including advanced statistical functions and 3D visualization.^96^ *ImageJ (Fiji)* is another widely-used open-source platform that offers powerful image processing capabilities with numerous plugins specifically developed for microscopy data analysis.^97^ Commercial software suites are proprietary and offer analysis platforms optimized for their respective instruments, often including specialized functions for particular measurement modes. *MountainsSPIP* from Digital Surf provides powerful 3D visualization and analysis tools, while also offering compatibility with various AFM manufacturers. For more customized analysis, many researchers turn to general scientific computing platforms such as *MATLAB, Python*, or *Igor Pro*, which offer flexibility for implementing custom analysis routines. Open-source projects like *AtomicJ* and *TopoStats* are gaining popularity, especially for batch processing and automated analysis of large datasets.^98^^,^^99^ The choice of software often depends on the specific analysis needs, data format compatibility, and users’ familiarity with different platforms.
(5) *Statistical analysis*: When reporting average values (e.g., mean modulus), include measures of variability (standard deviation) and the number of measurements. For heterogeneous samples, consider reporting distributions rather than single average values.
(6) *Raw data availability*: Consider making raw data available through repositories (e.g. GitHub/SI addition), allowing other researchers to perform independent analyses.

By carefully analyzing the specific data types generated by each AFM mode and following these reporting guidelines, researchers can maximize the value of their nanomechanical measurements. This guide ensures reliability and reproducibility of individual studies while advancing standardized protocols for AFM-based nanomechanical characterization of soft matter, enabling meaningful comparisons and meta-analyses across different research efforts.

## Conclusion

**Figure 6:**
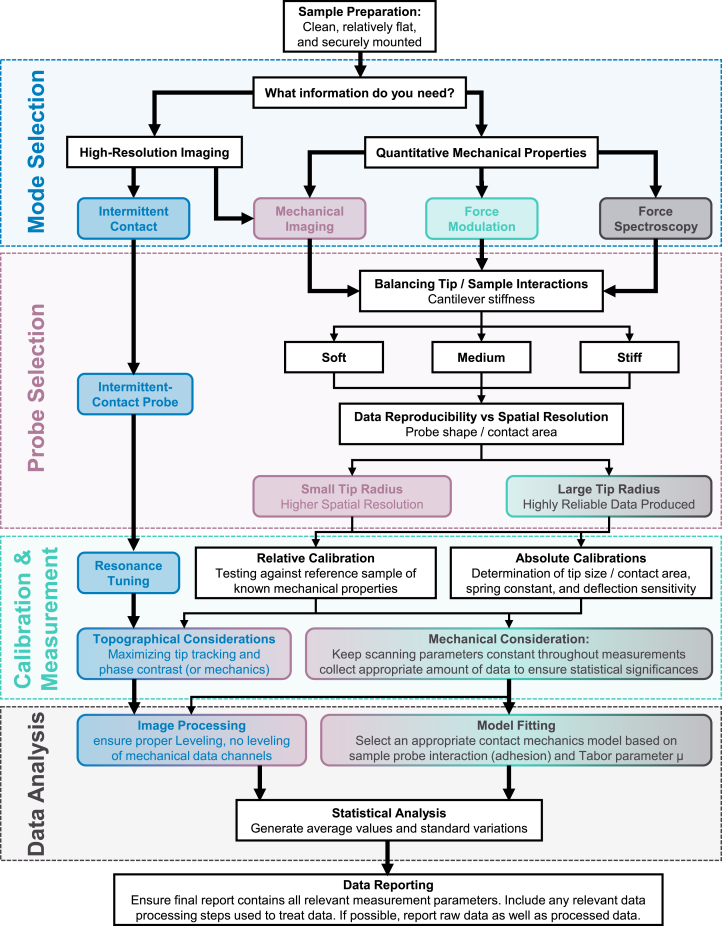
Comprehensive decision tree and workflow diagram for AFM nanomechanical measurements The flowchart illustrates the four key stages and modes discussed in this Primer.

Through a systematic approach to practical AFM measurement fundamentals, this Primer aims to establish a comprehensive methodology for achieving reproducible nanomechanical measurements on soft matter. By addressing key questions in the AFM measurement process—from mode selection and probe choice to calibration and data analysis—we provide a set of standardized guidelines that researchers can follow to enhance the consistency and comparability of their results. The entire workflow is summarized as a diagram in Figure 6, and a practical application of this workflow is provided in the supplemental information, which walks through a complete nanomechanical imaging experiment on a model polymer system.

By following the protocols and best practices outlined in this Primer, researchers can significantly improve the reproducibility of their AFM measurements. This standardization is crucial for advancing the field of nano-mechanics, enabling meaningful comparisons between studies, and accelerating progress in soft materials science and related disciplines.

Ultimately, this guide bridges the gap between theoretical knowledge and practical implementation in nanomechanical characterization of soft matter. It serves not only as a comprehensive resource for both novice and experienced AFM users but also as a call to action for the research community to adopt more rigorous and consistent practices in nanomechanical measurements. As the field continues to evolve, adherence to such standardized protocols will be essential for understanding and engineering soft materials at the nanoscale.

While this Primer is providing a comprehensive framework for current AFM nanomechanical measurements, the field continues to evolve rapidly. Emerging technologies promise to further enhance the capabilities and reliability of AFM-based nanomechanical characterization. An example of these developments are cantilevers with on-chip piezoelectric actuation and novel dual-sensing capabilities, which allow simultaneous measurement of tip displacement and tip force.^100^^,^^101^ Additionally, advances in artificial intelligence and machine learning are transforming data analysis in AFM, offering sophisticated approaches to model fitting, feature recognition, and the interpretation of complex contact mechanics.^102^ As these technologies mature, we anticipate even more precise and reproducible accessible nanomechanical characterization of soft materials.

## Acknowledgments

E.K. and A.L.R.F. acknowledge funding from the Stanford Graduate Fellowship (SGF). A.L.R.F, M.S., and E.Z. acknowledge support from the 10.13039/100000001National Science Foundation – Graduate Research Fellowship Program (NSF GFRP) under grant no. DGE-2146755. A.L.R.F. further acknowledges the National Consortium of Graduate Degrees for Minorities in Engineering (GEM) Fellowship and 10.13039/100005492Stanford University EDGE Fellowship Program. L.M. gratefully acknowledges funding through the Walter Benjamin Fellowship Program by the 10.13039/501100001659Deutsche Forschungsgemeinschaft (DFG 456522816). This work is in part supported by the Department of Defense Office of Navy Research (N00014-23-1-2446). Part of this work was performed at the Stanford Nano Shared Facilities (SNSF), supported by the 10.13039/100000001National Science Foundation under award ECCS-2026822. We greatly appreciate the comments and recommendations of Carina Yi Jing Lim and Dr. Ena Luis on the final manuscript.

## Author contributions

E.K., A.L.R.F., M.S., and E.Z. contributed equally to summarizing and developing the four main sections of the Primer. C.J.N. contributed through fruitful discussions and editing of the manuscript. Z.B. contributed to the improvement of the final document. L.M. guided the overall progress, performed sample measurements, contributed to the conceptualization of the document, and conducted final editing.

## Declaration of interests

The authors declare no competing interests.

## Supplemental information

Document S1. Guide on applying the principles outlined in this Primer, Figures S1–S9, and Table S1
