# Supplementary material for: A guide for nanomechanical characterization of soft matter via AFM: From mode selection to data reporting
Source: STAR Protoc. 2025 May 29;6(2):103809. doi: 10.1016/j.xpro.2025.103809 (PMC12166430; doi:10.1016/j.xpro.2025.103809)
Supplement: Document S1. Guide on applying the principles outlined in this Primer, Figures S1–S9, and Table S1 [file mmc1.pdf]

Guide on applying the principles of this Primer

A guide for nanomechanical characterization of soft matter via AFM: from mode selection to data reporting

Eunyoung Kim,[1] Alexandra L. Ramos Figueroa,[2] Max Schrock,[3] Elizabeth Zhang,[4] Christina J. Newcomb,[5] Zhenan Bao,[2] Lukas Michalek[2]*

[1]Department of Mechanical Engineering, Stanford University, Stanford, CA, 94305 USA
[2]Department of Chemical Engineering, Stanford University, Stanford, CA, 94305 USA
[3]Department of Chemistry, Stanford University, Stanford, CA, 94305 USA
[4]Department of Materials Science and Engineering, Stanford University, Stanford, CA, 94305 USA
[5]Stanford Nano Shared Facilities, Stanford University, Stanford, CA, 94305 USA

*Correspondence: lukasmi@stanford.edu

This supporting information provides a step-by-step guide on how to apply the principles outlined in the primer, using a model polymer blend system as an example. This protocol will walk through each decision point in the AFM measurement workflow – from sample preparation, mode selection, probe selection to calibration procedures and data analysis. By following one specific sample through the entire characterization process, this guide aims to illustrate the practical considerations, address common challenges, and recommend solutions that researchers may encounter when characterizing soft materials using AFM. This step-by-step approach serves as a reference for both novice and experienced AFM users, ensuring standardized procedures for reliable nanomechanical measurements. The workflow can be found as a summary in Figure 6 of the primer.

1. Model system

For this practical demonstration, we have selected a model system from the field of stretchable electronics: an in-situ rubber matrix (iRUM) formulation designed for dielectric applications by Zheng et al.[1] Specifically, we examine a two-component system consisting of polystyrene-block-poly(ethylene-co-butylene)-block-polystyrene (SEBS) and perfluorophenyl azide (PFPA) end-capped hydrogenated-polybutadiene (BH). This system represents an ideal case study for AFM nanomechanical characterization because of its technological relevance and complex phase behavior. The BH component functions as a crosslinking agent, creating a highly crosslinked network within the SEBS matrix upon thermal activation. The resulting crosslinked material provides both elasticity and solvent resistance, which are essential for practical applications in stretchable electronics. The phase separation and aggregation between these components and the mechanical contrast makes BH/SEBS an excellent candidate for demonstrating the AFM characterization workflow outlined in this primer.

2. Sample preparation

The preparation of the BH/SEBS blend films follows a controlled protocol to ensure reproducible and high-quality samples for AFM characterization. First, individual stock solutions of SEBS (80 mg/mL) and BH (100 mg/mL) were prepared using anhydrous toluene as the solvent. These solutions were filtered to remove particles or larger impurities that could introduce artifacts in the following AFM measurements. The filtered solutions were then mixed in a 1:1 volume ratio to create the BH/SEBS blend. To ensure proper dissolution and mixing, the combined solution was heated to 85°C under nitrogen atmosphere with continuous stirring for several hours. The resulting homogeneous solution was spin-coated onto highly doped silicon substrates (approximately 2×2 cm in size) at 1000 r.p.m. for 1 minute. The use of polished silicon wafers provides a flat substrate that minimizes any topographical contributions from the underlying surface. After deposition, the films were thermally annealed at 200°C for 1.5 hours in a nitrogen atmosphere to activate the crosslinking reactions. This annealing step is critical for forming the desired crosslinked network structure within the film. The resulting

films exhibit a thickness of approximately 1 μm, which is sufficient to prevent substrate effects from influencing nanomechanical measurements. When characterizing thin polymer films by AFM, maintaining sufficient film thickness is essential, as substrate influences can significantly alter the measured mechanical properties when indentation depths approach a significant fraction of the film thickness (as a rule of thumb usually less than 10% indentation of total film thickness).

3. Mode selection

Following the decision-making framework presented in Figure 3 of the primer, we carefully considered the specific requirements for characterizing the BH/SEBS blend system. The primary objective for the model system was to obtain both quantitative mechanical property measurements and high-resolution spatial information to understand the complex phase morphology. Given these requirements, nanomechanical imaging emerged as the optimal choice (see **Figure S1**). This mode provides quantitative mechanical property maps while maintaining sufficient spatial resolution to distinguish between the various phases present in our sample. The complexity of the BH/SEBS system arises from multiple levels of phase separation: (1) the primary phase separation between BH and SEBS components, (2) the inherent microphase separation within SEBS itself due to its block copolymer nature (polystyrene and poly(ethylene-co-butylene) domains), and (3) potential aggregation resulting from the annealing process. While intermittent contact mode would offer better spatial resolution, it lacks the quantitative mechanical information needed to fully characterize the BH/SEBS system. Conversely, force modulation would offer excellent measurements of viscoelastic properties, but at a significantly reduced spatial resolution, which could result in missing important morphological features. Force spectroscopy, though highly quantitative, would require an impractical number of measurement points to adequately capture the complex phase morphology. Nanomechanical imaging strikes the optimal balance, offering simultaneous topographical and mechanical mapping with sufficient spatial resolution to resolve the multi-scale phase features while providing the quantitative mechanical data necessary to distinguish between different regions.

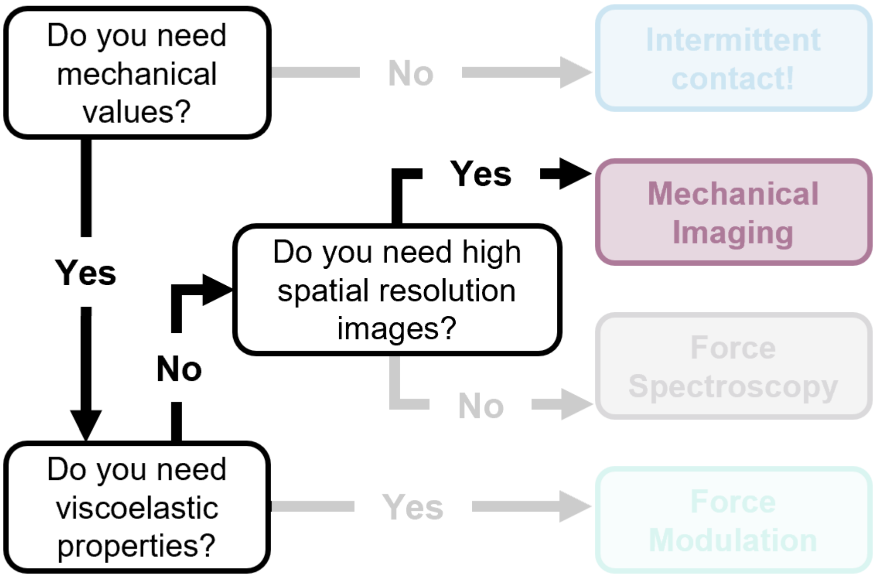

Figure S1: Mode selection decision tree highlighting nanomechanical imaging as the optimal choice for the BH/SEBS blend system.

In this example, a *Bruker Dimension Icon* AFM system equipped with a *Nanoscope V* controller with access to the *PeakForce-Quantitative Nanomechanical Measurement* (PF-QNM) mode was used. This implementation of nanomechanical imaging offers controlled force curves at a frequency of 0.25 – 2 kHz for rapid acquisition of typically the following data channels: Height, PeakForce Error, Stiffness/Modulus, Log(Stiffness), Adhesion, Indentation and Dissipation. It is worth noting that equivalent nanomechanical imaging capabilities are available on other commercial AFM platforms under different proprietary names, such as *Fast Force Mapping* on *Oxford Instruments/Asylum Research* systems, *PinPoint* measurements on *Park Systems* instruments, *Off-Resonance*

Tapping/WaveMode on *Nanosurf* AFMs, and similar implementations on other AFMs. While the specific software interfaces and terminology may differ between platforms, the fundamental principles of nanomechanical imaging discussed in our primer remain applicable across these various systems.

4. Probe Selection

For characterizing the BH/SEBS blend system, SCANASYST-AIR cantilevers from *Bruker AFM Probes* were selected as an optimal probe choice based on consideration of the sample's mechanical properties and morphological features. The nominal spring constant of **0.4 N/m** and nominal tip radius of **2 nm** is well-matched to the effective sample stiffness k_{eff} expected from the BH/SEBS system, which exhibits elastic moduli in the range of 5-**100 MPa**.

$$k_{eff} \approx 2Ea = 2 \cdot 100\ \mathrm{MPa} \cdot 2\ \mathrm{nm} = 2 \cdot 10^{8}\mathrm{N/m^2} \cdot 2 \cdot 10^{-9}\mathrm{m} = 0.4\ \mathrm{N/m}$$

Matching the values of cantilever spring constant (k) with sample stiffness (k_{eff}) provides sufficient deflection sensitivity to measure variations between different phases using an acceptable dynamic range of the detector. Mismatch can result in (1) sample damage and unreliable mechanical data if the cantilever is too stiff for a soft sample ($k_{eff} << k$) or (2) no sample deformation if sample is too stiff compared to the cantilever spring constant ($k_{eff} >> k$). Additionally, the sharp tip radius of 2 nm provides high spatial resolution – the boundaries between polystyrene and poly(ethylene-co-butylene) domains can be resolved with feature sizes on the order of tens of nanometers. The SCANASYST-AIR cantilevers also offer excellent stability during nanomechanical imaging, enabling consistent force control throughout the measurement process. Furthermore, these cantilevers provide a good balance between mechanical property mapping capabilities and topographical tracking, which is particularly important for this heterogeneous blend system where variations in both height and mechanical properties are expected to occur simultaneously across the sample surface. In the table below the typical ranges for probe parameters can be seen for nanomechanical imaging.

Table S1: Typical AFM probe parameter ranges for the nanomechanical imaging mode.

**AFM Mode**	Spring Constant / N/m	Resonance Frequency / kHz	Tip Radius / nm
Intermittent contact	~ 5 - 50	~ 150 - 400	~ 1 - 10
**Nanomechanical imaging**	**~ 0.1 - 5**	**~ 15 - 150**	**~ 1 - 20**
Force modulation	~ 1 - 40	~ 40 - 200	~ 8 - 100
Force spectroscopy	~ 0.05 - 15	~ 6 - 150	~ 50 - 5000

5. Calibration Procedure

The quantitative nature of nanomechanical imaging requires calibration of both cantilever mechanics and tip geometry.

Cantilever Calibration

We began with determination of the cantilever spring constant through thermal tuning, which measures the thermal noise spectrum of the cantilever to calculate its spring constant based on the equipartition theorem (**Figure S2A**). This was followed by measuring the deflection sensitivity by performing force curves against a hard reference surface (**Figure S2B**), in our case a sapphire reference sample from *Bruker*, though clean silicon substrates or glass slides would also provide sufficiently rigid surfaces for this calibration step.

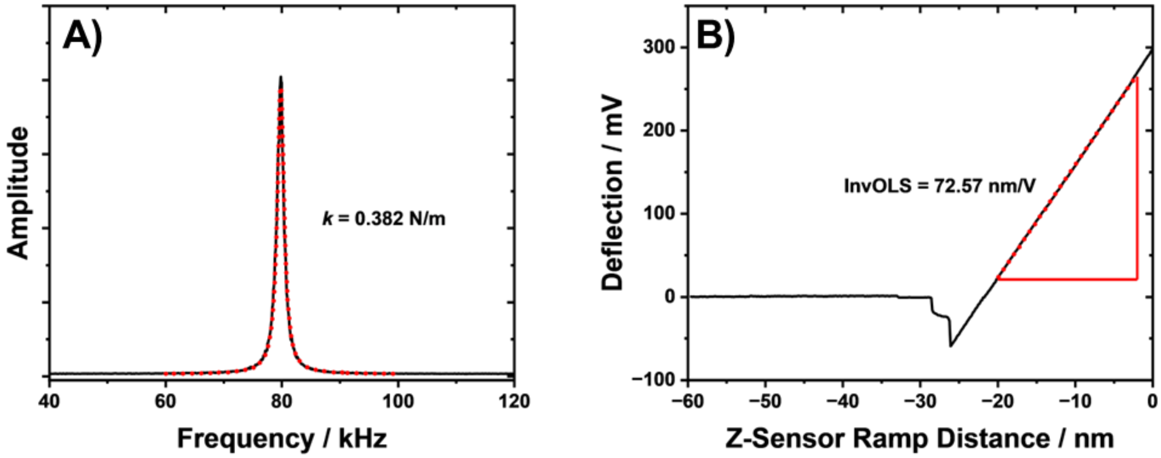

Figure S2: A) Thermal tune and B) Deflection-Z-sensor spectroscopy on Sapphire of SCANASYST-AIR cantilever.

Blind Tip Radius Estimation – Absolute & Relative Measurements

For tip radius estimation, we employed both absolute and relative calibration methods to ensure accuracy of the quantitative mechanical properties. First, we used a Titanium roughness reference sample from *Bruker* featuring numerous sharp grain boundaries, which allow the calculation of the tip features. The acquired image was analyzed using *Bruker's Nanoscope Analysis 3.0* software with the Tip Qualification feature (an exact walk through of each step can be found in the manufacturers handbook) to extract the tip geometry from the image features. Typical measurement parameters for the roughness reference sample are:

- PF-Frequency **2 kHz**	- Amplitude **30nm**	- Set-Point **0.5 nN**	- Gain ~**10**
- Scan Size **1.5 µm**	- Scan Rate **0.3 Hz**	- Lines **512x512**	

However, these parameters will vary depending on the AFM probe. Below you can see the resulting height image (Figure S3). The *Nanoscope Analysis 3.0* software estimates a tip radius of **r = 1.6 nm**.

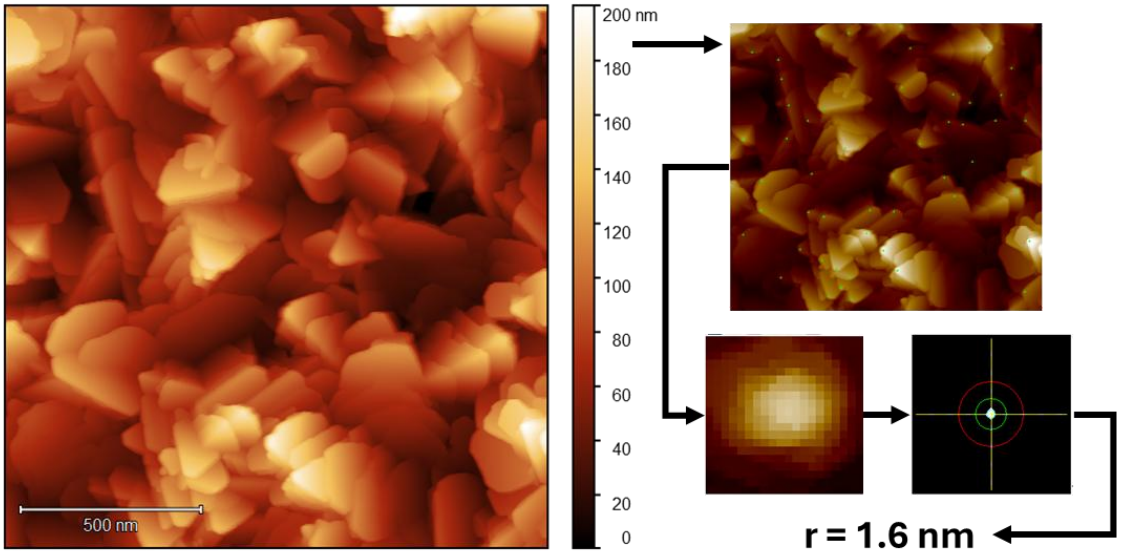

Figure S3: Height image of titanium roughness reference and absolute tip radius estimation via *Nanoscope Analysis 3.0*.

To obtain a more comprehensive three-dimensional representation of the tip shape, we also performed blind tip estimation using *Gwyddion* software (under Data Process → SPM Modes → Tip, applied to the Height channel of the acquired roughness reference). Due to the high roughness of the reference sample the noise suppression threshold was increased to ~ 5 nm. For the sake of demonstration, we performed the blind estimation for the maximum size (150x150 pixels). To avoid excessive computational time, we recommend to run the estimation procedure on a smaller size (~ 50x50 pixels) first and increase as needed. Below is the blind estimated tip

depicted with schematic representation of the tip geometry and two height profiles along the vertical and horizontal axis.

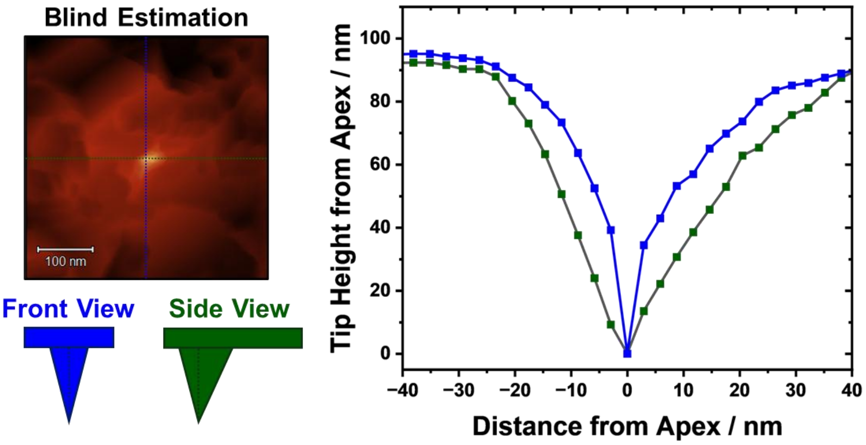

Figure S4: Blind tip estimation employing the height channel of the titanium roughness reference using *Gwyddion*.

We conducted a relative estimation of the tip size using a PDMS reference sample with a known elastic modulus of 3.5 MPa (*Bruker* standard). This approach accounts for the increased contact area that results when indenting further into a softer material, compared to harder samples. To account for this, the effective tip radius is typically adjusted to higher values in a stepwise manner using the measurement software (*Nanoscope 9.7* for our measurements). Increasing the tip-radius leads to a reduction of the measured modulus value. As shown in **Figure S5** when the modulus of the 3.5 MPa PDMS standard is measured based on the absolute measured tip radius of r = 1.6 nm the values are nearly 10 times higher than expected. When the tip radius is increased to r = 15 nm, the measured modulus falls within the expected range of 3.5 MPa. This result demonstrates that the absolute method provides an initial estimate, the relative method is more effective when a standard with a modulus range similar to the actual samples is available, which can be explained by the differences in indentation depth (indicated as dotted lines in the reconstructed tip in Figure S5) In the Data Analysis section, we will compare the differences between these two calibration methods using the BH/SEBS model system.

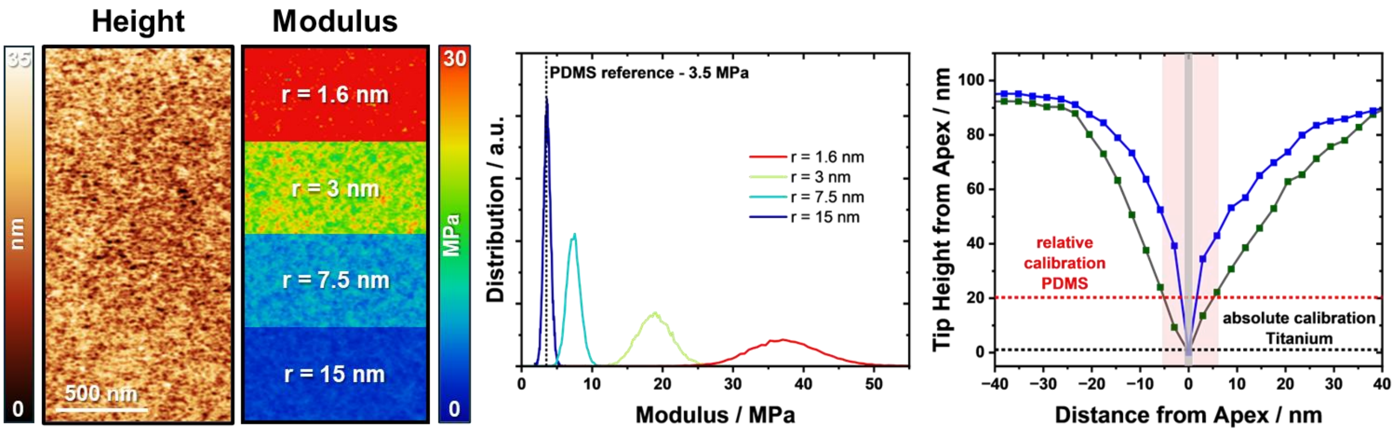

Figure S5: PDMS reference sample with a modulus of 3.5 MPa using different tip radius values. Difference of absolute vs relative tip radius estimations are shown on the reconstructed tip.

6. Measurement Settings and Optimization

Optimizing measurement parameters for the BH/SEBS blend system requires a balance between resolution, sensitivity, and scan speed to capture the complex morphological and mechanical features without introducing artifacts.

Image Quality Parameters
As general advice, a systematic approach to parameter adjustment can significantly improve both image quality and measurement efficiency. Always begin by setting conservative values:

- **moderate scan rates** (0.5 Hz),
- **intermediate force setpoints** (0.5-1 nN)
- **standard gains** (~ 10 for PF-QNM)
- **high frequency and amplitude** (2 kHz, 150nm),

It is always best to refine parameters sequentially. To improve image quality, increase the setpoint force slightly to enhance signal-to-noise ratio while watching for sample deformation. Optimize the feedback gains, increase until oscillations appear, then reduce slightly to find the optimal balance between good tracking and scan speed. Reduce amplitude as low as possible (still need to be able to be lifted from sample surface, overcoming adhesion) to reduce noise and other artifacts. For better image quality, decrease scan rates and increase pixel density (to avoid oversampling, pixel size should not be smaller than the tip size), this extends acquisition time.

Mechanical Measurement Parameters
Once engaged with the sample, the synchronization distance (the parameter controlling the portion of the force curve used for analysis) must be optimized. This involves adjusting the synchronization distance (usually given in %) until both the baseline region (before tip-sample contact) and the maximum force region are fully visible in the force curve, with the slopes of the approach and retract curves overlapping around the maximum force region. This alignment is essential for accurate mechanical property extraction, particularly for elastic modulus calculations, and requires optimization when the frequency or amplitude parameters are adjusted. For our viscoelastic polymer system, a synchronization distance of approximately ~ 30% provided optimal results. The typical force curves are depicted in **Figure S6**, which also shows the synchronization distance.

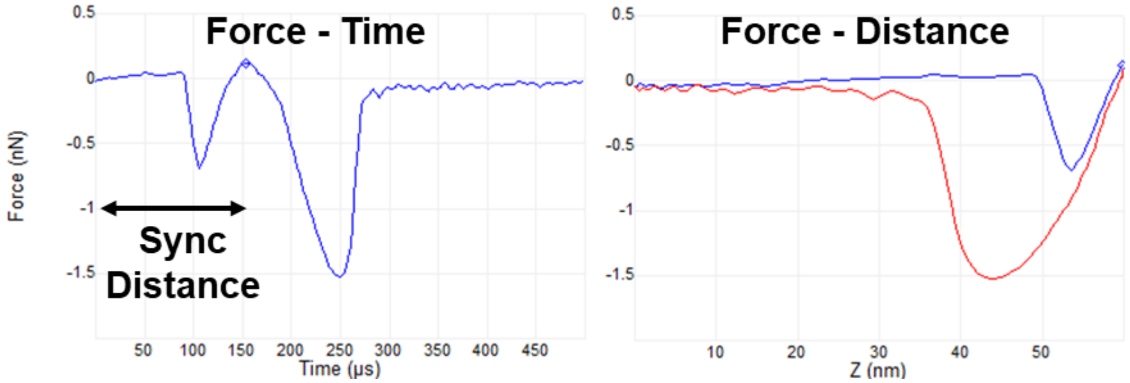

Figure S6: Synchronization "Sync" distance and Force-Time and Force-Distance curves of the BH/SEBS system.

For the model system, the following parameters were used: a low setpoint force **F ~ 200 pN**, a moderate scan speed **0.7 Hz**, a medium **gain 10** over a small scan area **2 μm**. We chose a PeakForce frequency of **2 kHz** and an amplitude of **30 nm**, as the sample is neither too sticky nor too rough (for sticky and/or rough samples the amplitude must be significantly increased). These settings provided sufficient indentation for mechanical contrast while minimizing sample deformation.

The sampling resolution was set to 256x256 pixels over the 2x2 μm area, providing a pixel size of approximately 10 nm, sufficient to resolve the microphase separation within the block copolymer structure. For mechanical property calculations, we selected the DMT model based on the moderate adhesion observed between the tip and sample surface (see Notes on contact mechanics in main publication).

Measurement Time
To reduce measurement time incrementally increase scan rates while monitoring force curve quality and potentially readjust the gains. Throughout this process, monitor multiple data channels simultaneously (height, modulus, adhesion, and indentation) to ensure that improvements in one aspect don't compromise others. The

optimal parameter set ultimately represents a balanced compromise between image quality, mechanical data accuracy, and practical time constraints.

7. Data Analysis

For the data analysis of our BH/SEBS system we are using the software *Gwyddion*. *Gwyddion* is a powerful open-source software for analyzing AFM data, offering robust tools for processing and visualizing nanomechanical properties.

Data Import and Leveling

The AFM data is opened by navigating to File → Open. *Gwyddion* automatically recognizes the file format and imports all channels. The first critical step in AFM data analysis is leveling and removal of sample tilt and scanner bow artifacts in the height channel. It is crucial to apply leveling only to the height channel while preserving raw mechanical property data. There are several different ways to level the data.[2] The most common are the "Level data by mean plane subtraction" or "Remove polynomial background", both found at Data Process → Level. For our BH/SEBS sample system we simply used the "Level data by mean plane subtraction" function, followed by setting the lowest value to zero by Data Process → Level → "Fix zero" (see **Figure S7**) If a scanner bow is visible a 2nd or 3rd-order polynomial leveling is a good choice. Additionally, if the piezo scanner accidently jumps/moves during your scan, you can Data Process → Correct Data → "Align rows" and "Remove Scars". Finally, the color-range can be adjusted in the Tools tab.

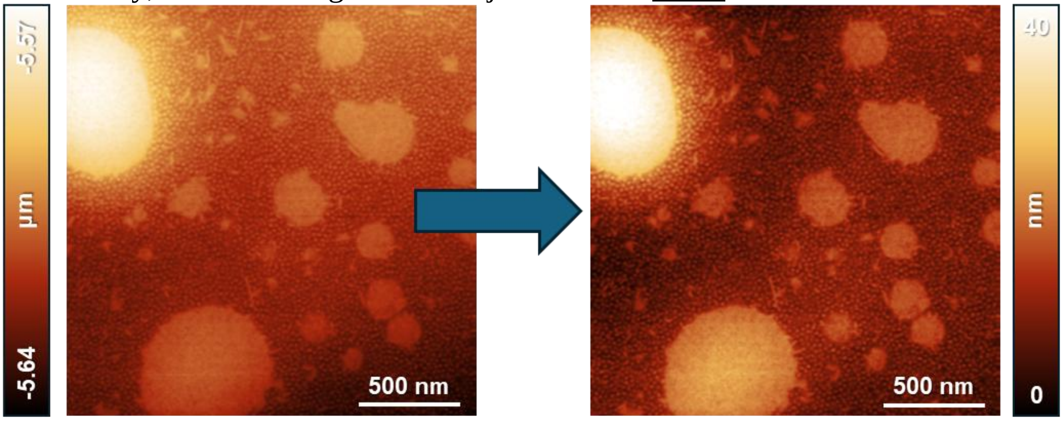

Figure S7: Initial to processed height image of the BH/SEBS system.

Analysis of Different Data Channels

The Nanomechanical mode (here PF-QNM) provides multiple data channels, each offering unique insight into the BH/SEBS blend's properties, which can be found when navigating to Info → Show Data Browser. We recorded following data channels: Height Sensor, PeakForce Error, DMT Modulus, logarithmic Modulus, Adhesion, Indentation, Dissipation and Height (the later two we are not shown the **Figure S8** below). We optimized the color range for each of the different channels. Furthermore, it would be possible to also change the false color pallet to highlight differences even further (this wasn't necessary for our model system). This can be done by a right click on the color range next to the image. For our model system we can clearly see the large-scale phase-separation of the BH component (the stiffer and sticky large spherical aggregates) and the smaller microphase separation of the SEBS block-co-polymer (best visible in the logModulus image).

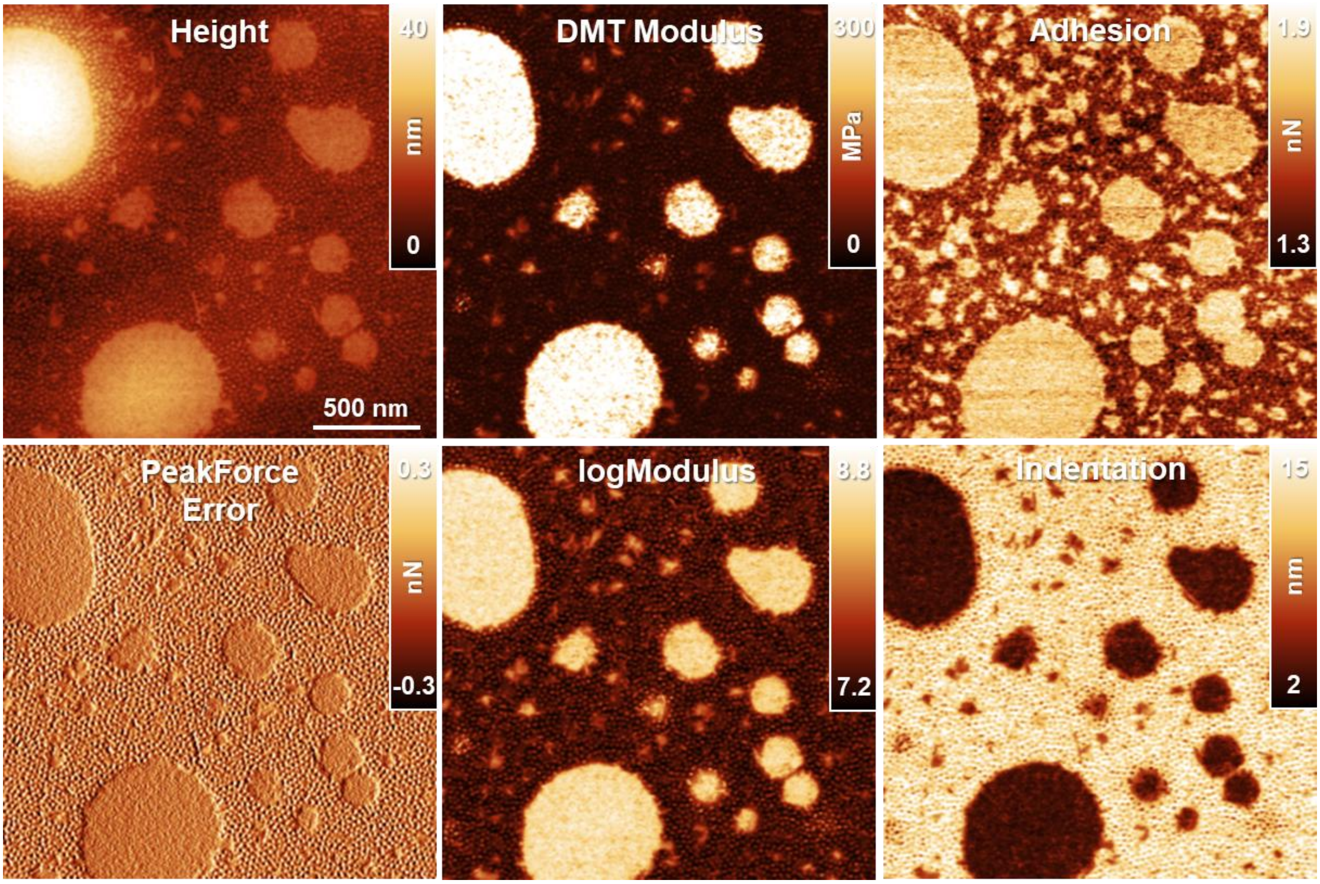

Figure S8: Different data channels of the BH/SEBS model system.

Statistical Analysis and Data Extraction

The AFM data for the BH/SEBS blend was systematically analyzed to extract quantitative mechanical properties from distinct phases. To comprehensively characterization the distribution of mechanical properties, the 1D statistical function (Tools → Calculate 1D statistical functions) was used to generate histograms. These histograms revealed multiple modulus populations that correspond to different structural components of the model system. As shown in **Figure S9**, measurements with different tip radii (1.6 nm and 15 nm) produced consistent phase identification, but the absolute modulus values were shifted, even though the indentation remained the same. This emphasizes the importance of proper tip characterization for accurate measurements. Additional insights were gained through line profile analysis (Data Process → Line Profile) across phase boundaries, revealing the spatial transition between domains. The indentation depth analysis further confirms that the measurements settings were appropriate, showing deeper indentation in softer phases while maintaining sufficient signal-to-noise ratio. This multi-parameter analysis approach enabled comprehensive characterization of the complex phase behavior in the BH/SEBS system, capturing both the statistical distribution of mechanical properties and their spatial organization within the blend. To further isolate different domains, it is possible to employ mask creation through Data Process → Mask → Create Mask, using thresholds based on a channel which has a strong contrast (like logModulus) that clearly distinguishes between phases in the blend and then distribute this to the other data channels by navigating to Data Process → Mask → Distribute.

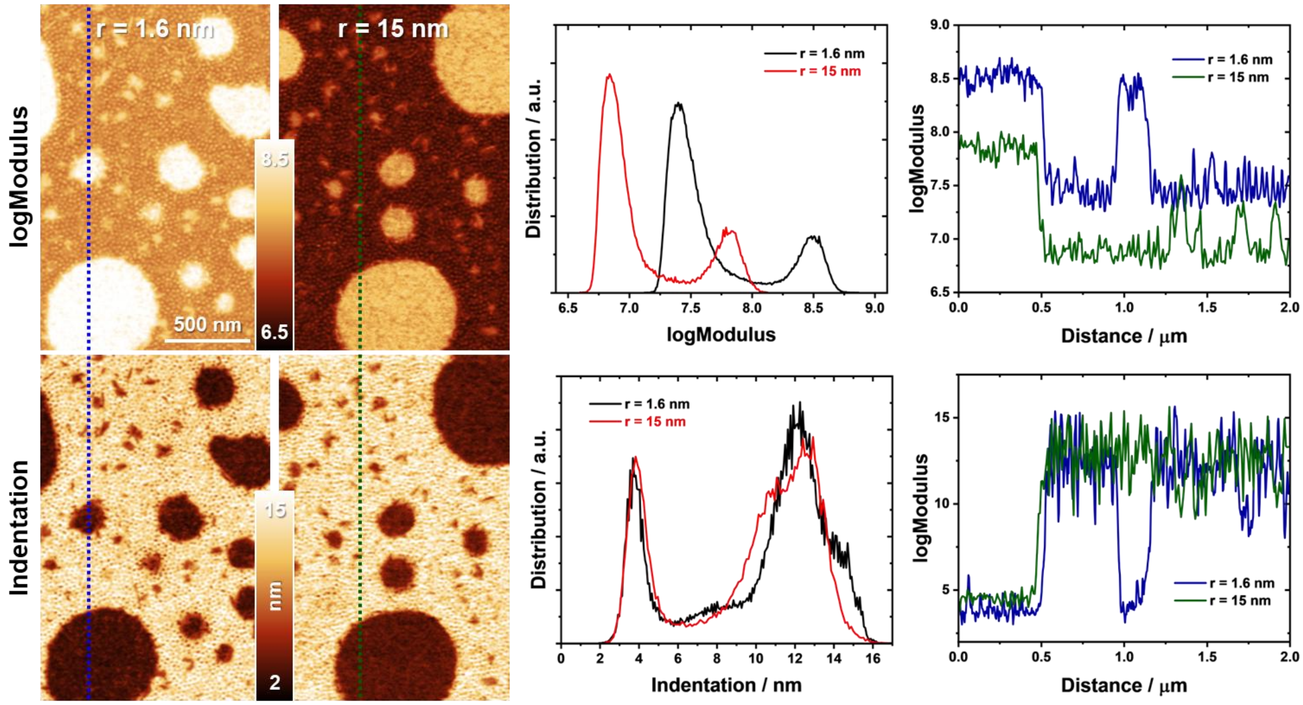

Figure S9: Indentation and logModulus for the absolute (r = 1.6 nm) and relative (r = 15 nm) calibration approach of the BH/SEBS model system, with extracted 1D statistical function and line profiles.

8. Reporting

When reporting the acquired AFM data in publications, comprehensive documentation of experimental parameters is essential for reproducibility. A standardized reporting format should include detailed specifications of the instrumentation, probe characteristics, calibration procedures, measurement settings, and data processing methods. An example for our model system would be: *"Nanomechanical images were collected using a Bruker Dimension Icon AFM with a Nanoscope V controller. SCANASYST-AIR cantilevers with a nominal force constant of 0.4 N/m were calibrated by thermal tuning (k ~ 0.382 N/m). The tip radius was estimated as 1.6 nm using sapphire and Ti reference samples (or 15 nm on a PDMS 3.5 MPa samples). The experiments were performed at a setpoint of 200 pN, a PeakForce frequency of 2 kHz, and an amplitude of 30 nm. Images with a scan resolution of 256x256 pixels at a scan rate of 0.7 Hz were obtained. The measured nanomechanical imaging data and height profiles were processed using Gwyddion SPM software 2.63."*

9. References

1. Zheng, Y., Yu, Z., Zhang, S., Kong, X., Michaels, W., Wang, W., Chen, G., Liu, D., Lai, J.-C., Prine, N., et al. (2021). A molecular design approach towards elastic and multifunctional polymer electronics. Nat. Commun. *12*, 5701. https://doi.org/10.1038/s41467-021-25719-9.
2. Nečas, D., and Klapetek, P. (2017). Study of user influence in routine SPM data processing. Meas. Sci. Technol. *28*, 034014. https://doi.org/10.1088/1361-6501/28/3/034014.
